# Letter from Dr. Spohn

**Published:** 1896-03

**Authors:** Arthur E. Spohn

**Affiliations:** Corpus Christi, Texas


### Correspondence.

### Letter From Dr. Spohn.

CORPUS CHRISTI, TEXAS, Feb. 20, 1896.

*Editors Texas Medical Journal:*

I have just returned from an extended European tour, and of course, had the pleasure of visiting many of the numerous hospitals, which was quite interesting. I can not say that I have anything to report new or peculiar to any place; for medical discoveries and new methods are so promptly reproduced, and distributed by our excellent journals, that the profession in the most remote districts can, if properly supplied with the current literature, keep well abreast with what is going on in the world. Visiting foreign countries tend to make us better pleased with our own, and feel, at least, that we are equal to the best. Our hospitals are newer, and more conveniently arranged, so that one need not go abroad for instruction or clinical advantages. I was well received everywhere, and feel thankful for the many courtesies extended me by members of the profession, who always welcome their American visitors, and try to interest them in every possible manner. I think our surgical instruments and appliances are superior; probably due to the varied nationalities represented in the profession of America. Such varied experience generating new ideas and devices peculiar to ourselves, which when perfected by our skilled instrument manufacturers, leave us little to desire that can not be supplied at home. At the request of Messrs. Tiemann & Co., I wish to mention a new modification in surgical instruments, which I have been using several years, but has not before been published. This modification consists in having all instruments so marked down the back of the handle, that the operator can tell, the moment he takes the instrument in his hand, just where the cutting edge, curve, hook, etc., may be located. This is very necessary for currettes, retractors, knives, in fact, all instruments where such markings can designate curves, hooks, etc., not by merely marking a small projecting point, but grooves all the way down the handle. In visiting the leading instrument manufacturers of Europe, I failed to find any instruments thus marked; and upon calling their attention to this fact, the modification was considered one of great importance. I think Messrs. Tiemann & Co., of New York, are the only manufacturers adopting this method, which I suggested to them several years ago. I have frequently seen cases reported where a currette has penetrated the walls of the uterus, and am not surprised at such accident, where an opertor is using a sharp instrument within a cavity, where he can not tell where the cutting edge is located. Operating under such circumstances led me to request my instrument maker to mark all instruments. Yours,

ARTHUR E. SPOHN, M. D.

